# Draft Genome Sequence of the Methane-Oxidizing Bacterium “*Candidatus* Methylomonas sp. LWB” Isolated from Movile Cave

**DOI:** 10.1128/genomeA.01491-16

**Published:** 2017-01-19

**Authors:** Jason Stephenson, Deepak Kumaresan, Alexandra M. Hillebrand-Voiculescu, Elliot Brooks, Andrew S. Whiteley, J. Colin Murrell

**Affiliations:** a School of Life Sciences, University of Warwick, Coventry, United Kingdom; b School of Earth and Environment, The University of Western Australia, Crawley, Perth, Australia; c Department of Biospeleology and Karst Edaphobiology, Emil Racoviţă Institute of Speleology, Bucharest, Romania; d School of Environmental Sciences, University of East Anglia, Norwich, United Kingdom

## Abstract

We describe the draft genome sequence of “*Candidatus* Methylomonas sp. LWB” isolated from Movile Cave microbial mat samples. The genome contains both the soluble and particular methane monooxygenase; however, one of the putative particulate methane monooxygenase gene clusters is ordered *pmoABC* rather than in the canonical gene arrangement of *pmoCAB*.

## GENOME ANNOUNCEMENT

“*Candidatus* Methylomonas sp. LWB” was isolated from Movile Cave, Romania, a sealed, sulfidic karst system sustained by chemolithoautotrophic microorganisms (1–6). Earlier studies showed that methane-oxidizing bacteria were present and active in Movile Cave (7) leading to an effort to isolate and characterize methylotrophs from this extreme environment (8). “*Ca*. Methylomonas sp. LWB” formed pink colonies when grown on solid medium with methane as the sole carbon and energy source. Its genome was found to contain genes encoding both soluble and particulate forms of methane monooxygenase along with a putative second particulate methane monooxygenase gene cluster with an unconventional gene arrangement (*pmoABC*).

The “*Ca*. Methylomonas sp. LWB” genome was sequenced at the Earlham Institute (Norwich Research Park, Norwich, United Kingdom) using Illumina MiSeq technology. Data sets were created for both 150 bp and 250 bp paired-end reads and an assembly of the combined data sets was used to produce a draft genome scaffold. The assembled data were uploaded to the RAST website for annotation and analysis (http://rast.nmpdr.org). Further analysis was also carried out by uploading the assembled genome to the IMG genome analysis website (http://img.jgi.doe.gov/).

The data assembled into 102 contiguous sequences amounting to 5,365,682 bp, 87.1% of which was predicted to account for coding sequence with a G+C content of 55.9%. A total of 5,296 genes were predicted with 5,225 potential protein coding genes of which 3,552 have an assigned function. Seventy one RNA genes were identified including 3 × 5 S rRNA, 6 × 16 S rRNA, 9 × 23 S rRNA, and 53 × tRNA genes. A full suite of enzymes required for the oxidation of methane through to carbon dioxide was identified. It appears that “*Ca*. Methylomonas sp. LWB” assimilates formaldehyde via the ribulose monophosphate pathway common to other species of *Methylomonas* such as *M. methanica* MC09. Genes encoding dissimilatory nitrate and nitrite reductases, together with putative nitrogenase genes suggest the ability of “*Ca*. Methylomonas sp. LWB” to denitrify and fix N_2_ but these metabolic traits need to be verified experimentally.

### Accession number(s).

This whole-genome shotgun project has been deposited at GenBank under the accession no. MKMC00000000. The version described in this paper is version MKMC01000000.
